# Innate and Adaptive Immune Response to Pneumonia Virus of Mice in a Resistant and a Susceptible Mouse Strain

**DOI:** 10.3390/v5010295

**Published:** 2013-01-21

**Authors:** Ellen R. T. Watkiss, Pratima Shrivastava, Natasa Arsic, Susantha Gomis, Sylvia van Drunen Littel-van den Hurk

**Affiliations:** 1 VIDO-Intervac, University of Saskatchewan, 120 Veterinary Road, Saskatoon, Saskatchewan, S7N 5E3, Canada; E-Mails: erw847@mail.usask.ca (E.R.T.W.); prs592@mail.usask.ca (P.S.); natasa.arsic@usask.ca (N.A.); 2 Veterinary Microbiology, University of Saskatchewan, 52 Campus Drive, Saskatoon, Saskatchewan, S7N 5B4, Canada; 3 Veterinary Pathology, University of Saskatchewan, 52 Campus Drive, Saskatoon, Saskatchewan, S7N 5B4, Canada; E-Mail: susantha.gomis@usask.ca (S.G.); 4 Microbiology and Immunology, University of Saskatchewan, 107 Wiggins Road, Saskatoon, Saskatchewan, S7N 5E5, Canada

**Keywords:** PVM, Balb/c and C57Bl/6 mice, innate and adaptive immunity, immunopathogenesis

## Abstract

Respiratory syncytial virus (RSV) is the leading cause of infant bronchiolitis. The closely related pneumonia virus of mice (PVM) causes a similar immune-mediated disease in mice, which allows an analysis of host factors that lead to severe illness. This project was designed to compare the immune responses to lethal and sublethal doses of PVM strain 15 in Balb/c and C57Bl/6 mice. Balb/c mice responded to PVM infection with an earlier and stronger innate response that failed to control viral replication. Production of inflammatory cyto- and chemokines, as well as infiltration of neutrophils and IFN-γ secreting natural killer cells into the lungs, was more predominant in Balb/c mice. In contrast, C57Bl/6 mice were capable of suppressing both viral replication and innate inflammatory responses. After a sublethal infection, PVM-induced IFN-γ production by splenocytes was stronger early during infection and weaker at late time points in C57Bl/6 mice when compared to Balb/c mice. Furthermore, although the IgG levels were similar and the mucosal IgA titres lower, the virus neutralizing antibody titres were higher in C57Bl/6 mice than in Balb/c mice. Overall, the difference in susceptibility of these two strains appeared to be related not to an inherent T helper bias, but to the capacity of the C57Bl/6 mice to control both viral replication and the immune response elicited by PVM.

## 1. Introduction

Pneumonia virus of mice (PVM) is a natural pathogen in rodents that induces severe respiratory illness which is in many ways similar to severe respiratory syncytial virus (RSV) infection in infants [1,2]. RSV is one of the most important causes of infant mortality and hospitalization worldwide and causes more cases of viral pneumonia in infants than any other virus [3,4,5]. It is highly transmissible, spreading through communities rapidly and infecting most of the population by the age of one [6]. RSV can infect and reinfect individuals throughout life and causes mild cold-like symptoms in most healthy adults and children [6,7]. In infants, young children, and immune-compromised individuals, however, the virus is more likely to progress to the lower respiratory tract, often causing an infection severe enough to require lengthy hospitalization and sometimes causing death [8]. The factors involved in the development of severe RSV are largely unknown, for a number of reasons. RSV pathogenesis involves the complex interplay of immune cells in the lungs, resulting in an inadequate or misdirected response that damages lung tissue [9]. In addition, studies on very young and severely ill infants are difficult, especially those involving the lower respiratory tract. The use of animal models can overcome many of the hindrances associated with human studies by providing access to the site of replication in the lower respiratory tract. However, the pneumoviruses are highly species-specific, so natural host-pathogen pairs are preferred or, arguably, necessary to examine the immune-mediated mechanisms of severe pneumovirus infection [10,11]. 

In the past three decades, PVM has been evaluated as a potential murine model of natural severe RSV infection. PVM causes respiratory illness that varies in severity depending on the virus and mouse strain used and can replicate to a high titre from a low inoculum [12,13]. To date two strains of PVM have been characterized, PVM J3666 and PVM 15. These two strains differ significantly in select regions of the G protein and throughout the sequence of the SH glycoprotein [13,14]. PVM 15 has a genome of 14.8 KB, containing 10 genes which encode 12 proteins. The genome sequence is 99.7% identical to that of PVM J366. With exception of a 137-amino acid protein encoded by a second ORF in the P mRNA, all PVM proteins have a respective counterpart in RSV [14]. An earlier study indicated that the PVM strain 15 is replication-competent, but non-pathogenic in mice [13,15]. However, more recently Krempl *et al.* concluded that a PVM 15 strain available from ATCC is pathogenic in Balb/c mice to a similar degree as the J3666 strain and lacks the same mutation seen in the PVM 15 strain variant used earlier [14,16], thus mimicking the original PVM strain 15 isolated by Horsfall and Hahn [17]. They also clarify that the earlier reported attenuation of the PVM strain 15 in mice was specific for that particular preparation and that the attenuation was not indigenous [14,16]. The susceptibility of different inbred mouse strains to infection with PVM J3666 has been studied extensively [12]. However, this does not apply to PVM 15, so the present study is focused on the direct comparison of the pathogenesis of PVM 15 in Balb/c and C57Bl/6 mice.

Balb/c mice show classic Th2-biased responses to several intracellular pathogens, making them more susceptible to severe infections compared to C57Bl/6 mice, which tend to show protective Th1-biased responses [18,19,20,21]. Balb/c mice are also more susceptible to RSV-induced eosinophilia following priming with the RSV G protein [22,23]. The basis of this susceptibility to eosinophilia is dependent on genetic background, rather than the MHC haplotypes expressed in these strains, as Balb/b mice, which express the same MHC haplotypes as C57Bl/6 mice in the context of the Balb/c genetic background, were as susceptible to eosinophilia as the Balb/c strain [22]. Thus, genetic differences in Balb/c and C57Bl/6 mice could lead to very different responses following natural pneumovirus infection *in vivo*. 

The goal of this study was to understand the basis of the difference in susceptibility between Balb/c and C57Bl/6 mice to PVM infection by comparing their immune response to PVM 15 *in vivo*. To investigate the immune response and pathogenesis of PVM in Balb/c and C57Bl/6 mice, we inoculated mice with increasing doses of PVM 15 and compared the level of weight loss and viral replication, the expression of key inflammatory mediators in, and recruitment of cells to the lungs, and the adaptive immune responses following infection. Clinical disease and viral replication was enhanced in the Balb/c strain, which correlated to earlier production of inflammatory mediators and influx of immune cells, particularly neutrophils and natural killer (NK) cells, when compared to the C57Bl/6 strain. PVM 15 induced virtually no Th2-biased cytokines or CCL11 (eotaxin) in either strain, and eosinophils were not detected in the lungs of infected mice. The adaptive immune responses, however, developed more rapidly and appeared to be more protective in C57Bl/6 mice. 

## 2. Results and Discussion

### 2.1. Balb/c Mice Show Earlier and More Weight Loss, and Enhanced Virus Replication in the Lungs after PVM 15 Infection in Comparison with C57Bl/6 Mice

**Figure 1:**
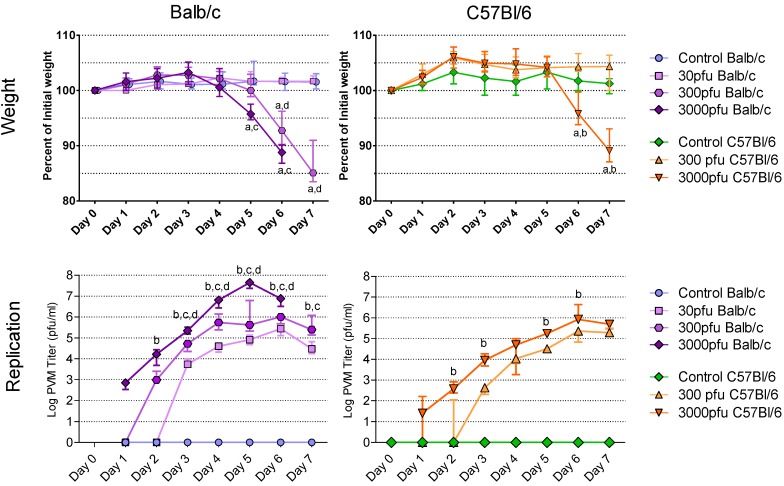
Weight loss and accumulation of virus in the lungs following infection with PVM 15 in Balb/c and C57Bl/6 mice. Five to six week-old Balb/c and C57Bl/6 mice were inoculated with medium, 30 pfu, 300 pfu, or 3000 pfu of PVM 15 and weighed daily for 7 days following infection, and 4 mice per group were sacrificed daily. (A-B) median weight for each group, expressed as a percentage of the starting weight, with error bars indicating the interquartile range. (C-D) median viral load for each group with error bars indicating the interquartile range. a: *p* <0.05 compared to control group; b. *p* <0.05 between 3000 and 300 pfu; c: *p* <0.05 between 3000 and 30 pfu; d: *p* <0.05 between 300 and 30 pfu. Differences in virus replication with the control groups are not shown.

As the first sign and best measure of clinical illness, the animals’ weights were recorded daily from day 0 to day 7 post infection (p.i.) (Figure 1A and B). Overall, Balb/c mice lost weight earlier than C57Bl/6 mice and succumbed to the PVM infection at a lower dose than the latter strain. No weight loss was observed in control mice, in Balb/c mice inoculated with 30 pfu of PVM, or in C57Bl/6 mice given 300 pfu or 30 pfu (data not shown) during the 7-day period after PVM challenge. Balb/c mice, however, lost weight as early as day 5 following inoculation with 300 pfu of PVM 15 and dropped to a median of 93% and 85% of their starting weight on days 6 and 7 p.i., respectively. At a dose of 3000 pfu, Balb/c mice began losing weight one day earlier, dropping to a significantly lower weight of 96% on day 5 and 89% on day 6 p.i.. On days 5 and 6 there was a significant difference in weight loss between Balb/c mice infected with 3000 pfu and those given 30 pfu, while the difference between the Balb/c mice given 300 and 30 pfu doses was significant on days 6 and 7. When given 3000 pfu, the C57Bl/6 mice showed the same degree of weight loss as Balb/c mice one day later, dropping to a median of 96% on day 6 and 89% on day 7 p.i. of their starting weight. The C57Bl/6 mice infected with 3000 pfu lost significantly more weight than those given 300 pfu on days 6 and 7 p.i.. Among the Balb/c mice challenged with 3000 pfu of PVM 15 all survived through day 5 p.i., while by day 6, 25% had died and by day 7 all had died. The C57Bl/6 mice challenged with 3000 pfu all survived through day 6, but 25% had died by day 7. All other mice survived.

The susceptibility of Balb/c and C57Bl/6 mice was further evaluated based on the level of viral replication in the lungs. At all three doses, Balb/c mice had a more rapid accumulation of virus in the lungs that reached an earlier peak followed by a clear decline (Figure 1C), while the C57Bl/6 strain displayed a slower increase in viral replication that reached a plateau on day 6 at both doses (Figure 1D). Indeed, PVM replication was so rapid in Balb/c mice that by days 3 and 4 p.i., the 30 pfu dose had replicated to similar titres as the 3000 pfu dose in C57Bl/6 mice, and by day 6 p.i. reached a peak that was equal to that of the 300 pfu C57Bl/6 mice. The amount of virus recovered from the lungs of infected mice was dose-dependent in the Balb/c strain at all times with the 3000 pfu dose causing more virus replication than the 300 and 30 pfu doses from days 2 through 7, and the 300 pfu dose more than the 30 pfu dose from days 3 through 6 p.i.. The 3000 pfu group of C57Bl/6 mice reached a significantly higher median viral titre than the 300 pfu group, except on days 4 and 7, when the C57Bl/6 mice infected with 300 or 3000 pfu had similar titres. A comparison of equivalent PVM 15 challenge doses in Balb/c and C47Bl/6 mice showed that 3000 pfu or 300 pfu produced a significantly higher viral load in the Balb/c mice than in the C57Bl/6 mice from days 2 through 6 p.i. (*p* < 0.05). Based on this direct comparison, PVM 15 causes more severe disease in Balb/c mice than in C57Bl/6 mice. 

Interestingly, Anh *et al.* [12] have characterized different strains of mice with respect to resistance to disease induced by ~1000 pfu of PVM J3666. Based on a combination of clinical, histological and virological parameters, the SJL mouse strain was most resistant followed by C57BL/6, BALB/c, C3HeN, DBA/2 and129/Sv strains. This is in agreement with the observations we made for PVM 15 in C57Bl/6 and Balb/c mice. 

### 2.2. Comparison of Lung Pathology in PVM-Infected Balb/c and C57Bl/6 Mice

**Figure 2:**
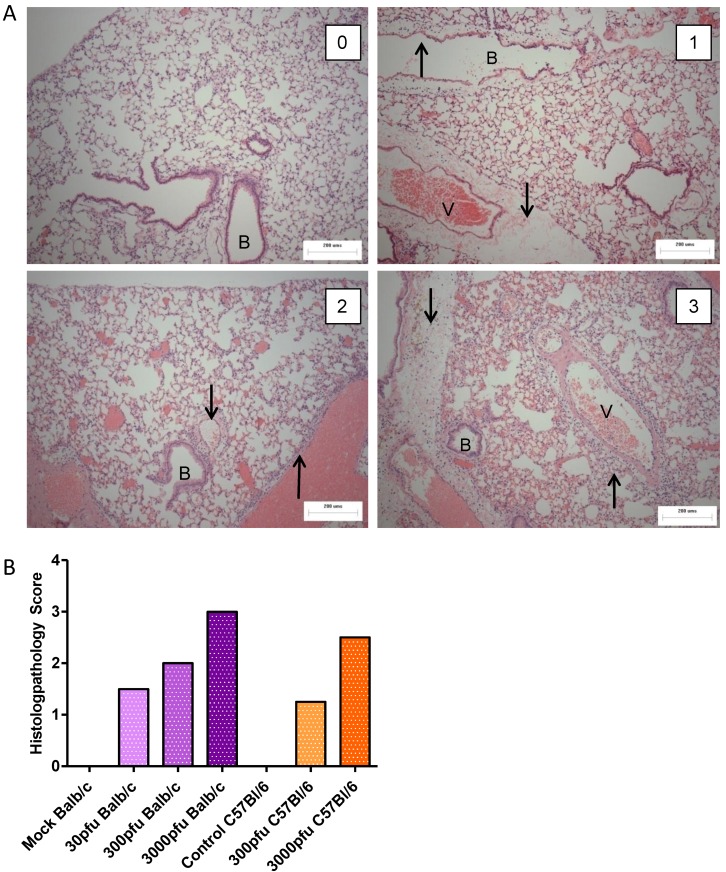
Histopathological analysis of PVM-infected mice. Five to six week-old Balb/c and C57Bl/6 mice were inoculated with medium, 30 pfu, 300 pfu, or 3000 pfu of PVM 15 and lungs were collected from four mice on day 6 p.i. for histopathological analysis. In (**A**), representative lung sections for animals scoring **0**, **1**, **2**, and **3** are shown, with the upward arrows (↑) indicating infiltrating inflammatory cells and the downward arrows (↓) indicating oedema in the tissue. The bronchiole is labeled with the letter B and the blood vessel with V. Scores were given on the basis of the severity and dissemination of the lesions visible in duplicate lung sections, and median values are shown for each group (**B**).

To assess the level of lung pathology induced by different doses of PVM in Balb/c and C57Bl/6 mice, lungs were processed for histopathological analysis on day 6 p.i. (Figure 2). Mice in the control groups had a score of zero, or normal lungs (Figure 2A, upper left panel), had few cells dispersed throughout the alveolar space, and the airway epithelium was intact and free of fluid and infiltrating immune cells. Although there was no significant difference in lung score between PVM-infected groups, there was a trend towards a dose-dependent increase in lung score for both strains of mice (Figure 2B). The C57Bl/6 mice given 300 pfu had a score close to 1, which indicates a localized, mild inflammation of the peribronchiolar and perivascular space involving fluid accumulation with few infiltrating immune cells (Figure 2A, upper right panel). The Balb/c mice inoculated with 30 or 300 pfu scored 1.5 to 2, which indicates multiple lesions or a single extensive lesion, the lesions being more severe, with higher numbers of infiltrating cells in the inflamed tissue and the alveolar space (Figure 2A, lower left panel). The 3000 pfu C57Bl/6 group had a median score of 2.5, while the 3000 pfu Balb/c group was scored as 3, indicating broadly dispersed lesions with cellular infiltrates in the alveolar space and surrounding tissues. The severity of the lesion is evident by the presence of inflammatory cells in the alveolar space and surrounding the blood vessel and bronchiole. There is little air space left in the lung, as much cell debris has accumulated in the alveoli (Figure 2A, lower right panel). These data show that overall, the level of lung pathology tended to be lower in the C57Bl/6 mice than in the Balb/c mice when infected with the same dose of PVM. 

### 2.3. PVM 15 Induces Earlier Transcription of Chemokines and Cytokines in Balb/c Mice When Compared to C57Bl/6 Mice

**Figure 3:**
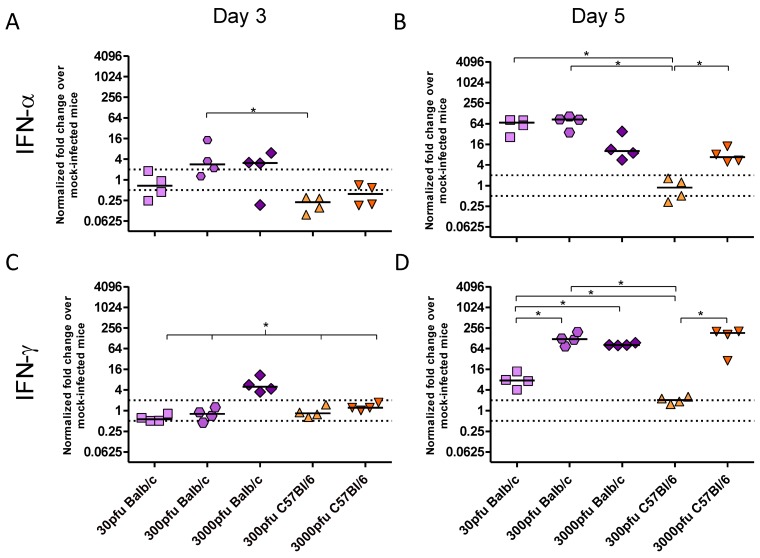
Cytokine expression by Balb/c and C57Bl/6 mice following infection with PVM 15. Five to six week-old Balb/c and C57Bl/6 mice were inoculated with medium, 30 pfu, 300 pfu, or 3000 pfu of PVM 15 and lungs were collected from four mice per group on days 3 (**A** and **C**) and 5 p.i. (**B** and **D**). Expression levels of IFN-α (**A** and **B**) and IFN-γ (**C** and **D**) transcripts were calculated using the Bio-Rad analysis software (Bio-Rad CFX Manager Version 2.0), normalized against the expression of both β-actin and GAPDH housekeeping genes, and expressed as the normalized fold-change over mock-infected control animals euthanized on the same day p.i.. Each data point represents a single animal and the line represents the group median. *, *p* <0.05.

**Figure 4:**
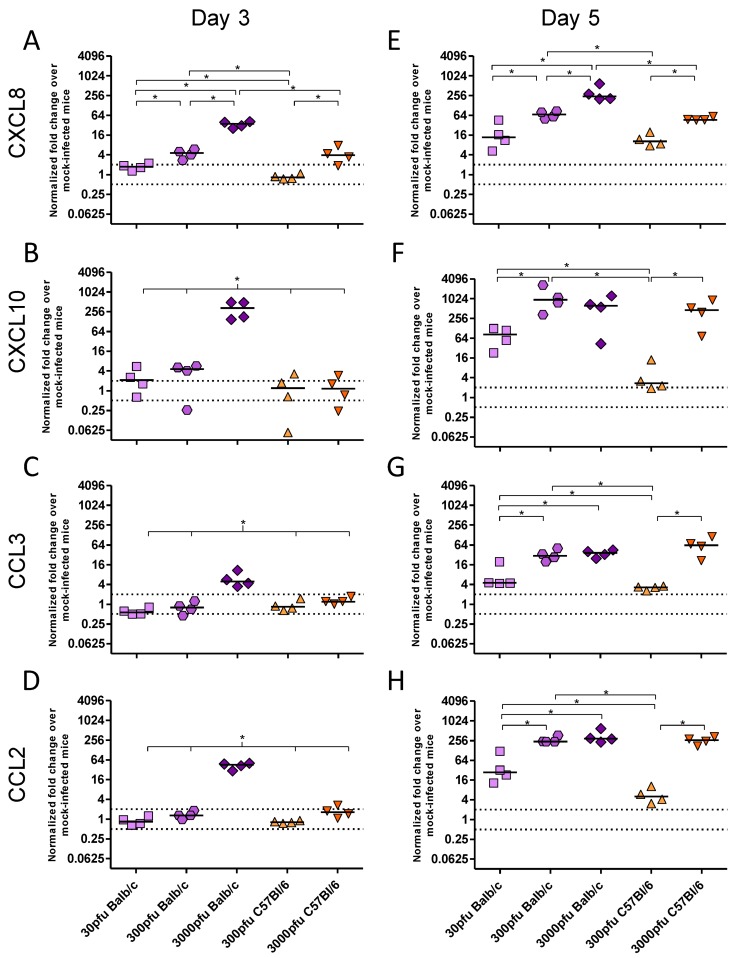
Chemokine expression by Balb/c and C57Bl/6 mice following infection with PVM 15. Five to six week-old Balb/c and C57Bl/6 mice were inoculated with medium, 30 pfu, 300 pfu, or 3000 pfu of PVM 15 and lungs were collected from four mice per group on days 3 (**A**–**D**) and 5 p.i. (**E**–**H**). The expression levels of CXCL8 (**A** and **E**), CXCL10 (**B** and **F**), CCL3 (**C** and **G**), and CCL2 (**D** and **H**) were calculated using the Bio-Rad analysis software (Bio-Rad CFX Manager Version 2.0), normalized against the expression of both β-actin and GAPDH housekeeping genes, and expressed as the normalized fold-change over mock-infected control animals euthanized on the same day p.i.. Each data point represents a single animal and the line represents the group median. *, *p* <0.05.

As there was a significant difference between PVM-infected Balb/c and C57Bl/6 mice in clinical disease, virus replication and lung pathology, the innate responses, in particular the production of key inflammatory cytokines and chemokines, were investigated. There was little to no upregulation of IFN-β, TNF-α, IL-4, IL-10, CCL5, or CCL11 upon infection with PVM (data not shown). In contrast, expression of IFN-α and IFN-γ (Figure 3), as well as CXCL8, CXCL10, CCL3, and CCL2 (Figure 4) changed significantly upon infection with PVM. 

There were three distinct patterns of mRNA expression following infection with PVM. IFN-α and CXCL8 showed unique profiles, while IFN-γ and the remaining chemokines had virtually identical patterns of upregulation. Strikingly, the C57Bl/6 mice showed virtually no upregulation of any chemokine or cytokine other than CXCL8 following infection with 300 pfu, in contrast to Balb/c mice which showed higher levels of all transcripts following inoculation with 300 pfu and even as little as 30 pfu of PVM 15. 

On day 3 p.i., IFN-α mRNA expression was not increased in any group, and, indeed, the C57Bl/6 mice appeared to have downregulated this cytokine. By day 5, however, the 3000 pfu dose of PVM 15 induced similar levels of IFN-α in the two mouse strains, while 30 and 300 pfu induced IFN-α upregulation only in Balb/c mice. Direct analysis of the role of type I IFN in PVM infection has only been carried out in C57Bl/6 mice with a deletion in the IFN-α/β receptor gene. Abrogation of type I IFN signalling during PVM J3666 infection led to decreased expression of IFN response genes, lower levels of IFN-α and IFN-β, and, intriguingly, a slightly enhanced survival time in C57Bl/6 mice given a lethal dose of 60 pfu [24]. This would agree with the lower degree of IFN-α expression and better survival we observed in PVM 15 infected C57Bl/6 mice in comparison to Balb/c mice. 

The CXC chemokine, CXCL8, was upregulated in a dose-dependent manner on both days 3 and 5 p.i., such that a 10-fold higher dose of PVM induced between 3 and 5-fold higher levels of mRNA upregulation in the Balb/c strain, and ~7-fold higher upregulation in the C57Bl/6 strain. IFN-γ and the remaining chemokines were upregulated in the 3000 pfu Balb/c group on day 3 p.i., and by day 5 they were expressed by all groups other than the 300 pfu C57Bl/6 group. The lethal doses of PVM induced similar mRNA upregulation of these proinflammatory mediators in Balb/c and C57Bl/6 mice by day 5, which was significantly higher than that in the 30 pfu Balb/c or the 300 pfu C57Bl/6 groups. Expression of CXCL10 was induced upon infection with PVM in a similar pattern to IFN-γ, which would be expected based on its role as an interferon response gene. Its mRNA upregulation was also similar to that of the CC chemokines, CCL2 and CCL3, with the three lethal doses showing a similar level of gene upregulation on day 5 p.i.. 

PVM J3666 pathogenesis is strongly governed by CCL3 production *in vivo*. Transgenic mice in the C57Bl/6 background that lack functional CCL3 or CCR1 were more susceptible to infection with PVM J3666, and had drastically reduced levels of neutrophil infiltration, with no eosinophils and very low levels of lymphocytes in the lungs upon infection [25,26]. The virus also replicated to a higher titre in these mice and they succumbed to infection earlier than non-transgenic littermates. However, blockade of CCL3 signalling by the CCR1/CCR5 receptor antagonist, Met-RANTES, resulted in decreased lung inflammation without affecting PVM replication in the lungs [25]. This is in agreement with our observation that C57Bl/6 mice, which responded to PVM 15 infection with delayed and reduced CCL3 expression as well as less neutrophil infiltration, also showed less disease and lung pathology when compared to Balb/c mice. 

Despite reports that CCL2 is one of the predominant chemokines upregulated during PVM infection, its exact role in PVM pathogenesis has not been defined yet [1]. CCL2 was significantly decreased by use of the antiviral ribavirin, suggesting that PVM replication stimulates its production *in vivo* [27]. Interestingly, in another study on age-related changes in susceptibility older mice showed decreased CCL2 production in response to PVM, which was associated with reduced disease presentation, despite similar viral replication [28]. This would support a role for the increased CCL2 expression in the enhanced pathogenesis in PVM 15-infected Balb/c mice. 

### 2.4. Balb/c Mice Experience Earlier and Enhanced Infiltration of Immune Cells Compared to C57Bl/6 Mice in Response to PVM Infection

**Figure 5:**
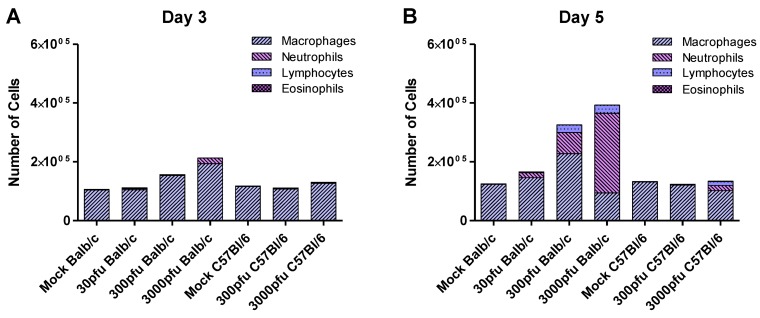
Infiltration of immune cells in Balb/c and C57Bl/6 mice in response to PVM infection. Five to six week-old Balb/c and C57Bl/6 mice were inoculated with medium, 30 pfu, or 300 pfu of PVM 15 and sacrificed on days 3 and 5 p.i.. Cells collected from the pooled lung washes of 4–6 animals were stained and analyzed for the presence of macrophages, neutrophils, lymphocytes , and eosinophils. The average number of these cells is shown, calculated based on the number of total cells collected from the group and the proportion of these cell populations in the lungs.

In order to determine the biological effects of the proinflammatory mediators reported above, we examined cell populations infiltrating the lungs of the infected mice (Figure 5). As expected from the earlier upregulation of proinflammatory mediators seen in Balb/c mice, this strain showed an earlier and stronger influx of cells into the lungs that was dominated by neutrophils. The degree of neutrophilia appeared to be dose-dependent in the Balb/c strain on day 5 p.i., while the lethal doses showed similar levels of lymphocyte infiltration. The C57Bl/6 mice, on the other hand, had little influx of cells by day 5 p.i., even at the highest dose of 3000 pfu. Comparing the two sublethal doses, the 30 pfu Balb/c group showed a low level of inflammation by day 5 p.i., while the 300 pfu C57Bl/6 group had essentially normal lung washes. There were few to no eosinophils in response to PVM 15 in either strain at any dose. In contrast, Domachowski *et al.* demonstrated that in response to PVM J3666, Balb/c mice showed a prominent eosinophilia, as early as 3 days after inoculation, while at later time points cell infiltration was dominated by neutrophils. In their study they did not find any up regulation of eosinophilic chemoattractant IL-5, CCL2, CCL11 or CCL5, but CCL3 was up regulated. Thus, pulmonary eosinophilia and production of CCL3 are prominent responses to infection with PVM J3666 [29]. 

Macrophages were the predominant population in the lungs at homeostasis. The number of macrophages tended to increase in the Balb/c mice on days 3 respectively 5 for the 3000 and 300 pfu doses of PVM, but remained stable in the C57Bl/6 mice. Rigaux *et al.* determined that depletion of alveolar macrophages in PVM J366-infected Balb/c mice resulted in prolonged survival, despite a slight but significant increase in viral replication [30]. This suggests that macrophages may also play a role in the enhanced pathology observed in PVM 15-infected Balb/c mice, although this would need to be further investigated. 

CXCL8 is produced in response to RSV infection of infants and is associated with disease severity and neutrophil degranulation products [31,32,33,34]. Although CXCL8 also is an important mediator of neutrophil recruitment following PVM infection, recent work on the contribution of CCL3 and IFN-γ to PVM pathogenesis suggests that these latter proinflammatory mediators may be as important, if not more so, than CXCL8 in recruiting neutrophils to the lungs of PVM-infected mice. Abrogation of IFN-γ or CCL3 signalling drastically reduced the number of neutrophils recruited to the lungs of PVM-infected C57Bl/6 mice [35]. In an IFN-γ^−/−^ Balb/c background, overexpression of CCL3 in the lungs was not sufficient to induce neutrophil recruitment to the lungs, despite the expression of its receptor on neutrophils in these mice [35]. When IFN-γ was administered to mice that overexpress CCL3 in the lungs, however, they rapidly developed neutrophilic inflammation and showed signs of clinical illness and weight loss similar to that experienced by PVM-infected mice. In our study, CCL3 and IFN-γ were (highly) upregulated by Balb/c mice upon infection with 30 or 300 pfu, while these chemokines were not expressed in C57Bl/6 mice given 300 pfu; furthermore, C57BL/6 mice had lower numbers of inflammatory cells, in particular neutrophils, in the lungs than Balb/c mice. These data agree with a role of CCL3 and IFN-γ in neutrophil recruitment, as well as the enhanced disease symptoms in PVM15-infected Balb/c mice.

### 2.5. Balb/c Mice Show an Increase in IFN-γ Producing NK Cells in the Lung Compared to C57Bl/6 Mice in Response to PVM Infection

**Figure 6:**
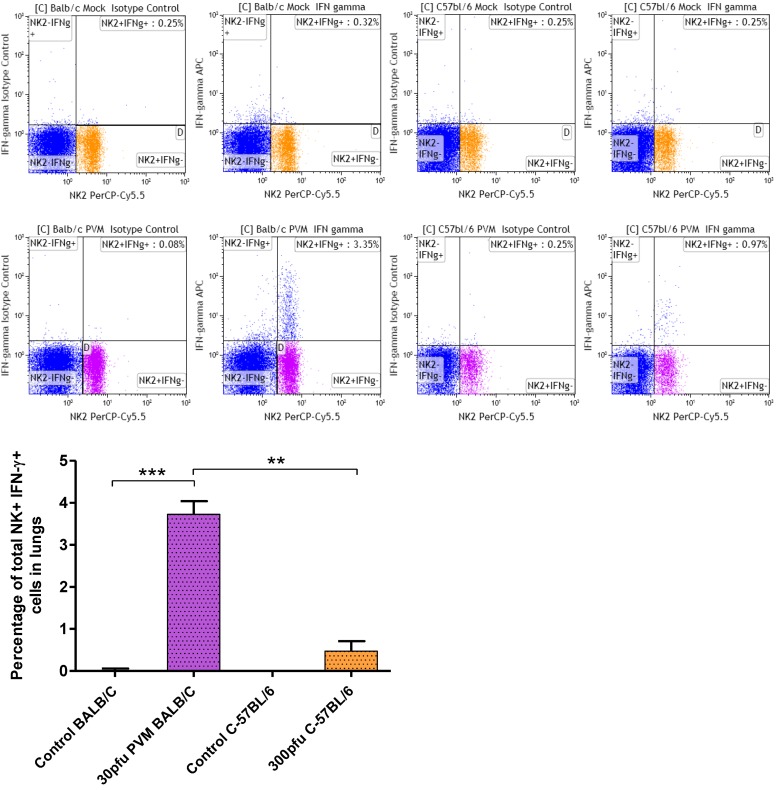
Percentage of IFN-γ secreting NK cells in lungs of Balb/c and C57Bl/6 mice in response to PVM infection. Five to six week old-mice were inoculated with medium (control), or 30 pfu (Balb/c), or 300 pfu (C57Bl/6) of PVM 15 and sacrificed on day 6 p.i.. Cells collected from pooled lungs of five animals were stained with anti-CD8a-FITC, anti-CD3-PE, anti-CD4-FITC, anti-CD335(NKp46)PerCP-Cy^TM^ 5.5 for surface markers, followed by permeabilization and fixation with BD Cytofix/Cytoperm^TM^ Plus and staining with anti-IFN-γ-APC. Flow cytometry was performed using a FACS Calibur (BD Biosciences), and data analysis was performed using Kaluza software. Median values with interquartile range are shown. **, *p* <0.01; ***, *p* <0.001.

As by day 5 p.i. IFN-γ and CXCL10 were significantly upregulated in the lungs, the population responsible for IFN-γ production was further characterized by dual staining and flow cytometry on day 6 p.i. While no IFN-γ secreting CD4^+^ or CD8^+^ T cells were found, a significant increase in IFN-γ secreting NK cells was observed in Balb/c mice, and a moderate increase in C57Bl/6 mice (Figure 6). The difference in IFN-γ producing NK cell in Balb/c and C57Bl/6 mice was significant, suggesting that NK cells contribute to lung pathology observed in Balb/c mice. Our results with respect to the absence of IFN-γ secreting T cell populations in lungs of PVM 15 infected mice agrees with previous reports by Claassen *et al.* using PVM J3666, in which authors demonstrated that during primary and secondary infection CD8 T cells are functionally restricted in IFN-γ production. They speculated that PVM infection results in effector T cell inactivation as reported in case of RSV infection [36].

Several chemokines are reported to act as chemoattractants to NK cells, including CCL3, CCL2, CXCL8, CCL5, and CXCL10 [37,38,39]. NK cells have been found to accumulate both after RSV [40] and PVM [41] infection of Balb/c mice, and to contribute to lung immune injury after RSV infection [40]. In our study, CCL3, CCL2, CXCL8 and CXCL10 were all upregulated after PVM infection specifically in Balb/c mice, CXCL8 as early as three days after infection, which agrees with the greater number of NK cells in Balb/c mice. The increased numbers of IFN-γ secreting NK cells in Balb/c mice when compared to C57Bl/6 mice further support a role for NK cells and IFN-γ in the enhanced disease observed in PVM-infected Balb/c mice. 

### 2.6. Balb/c and C57Bl/6 Mice Develop Similar, Th1-Biased Adaptive Immune Responses to a Sublethal Dose of PVM

**Figure 7:**
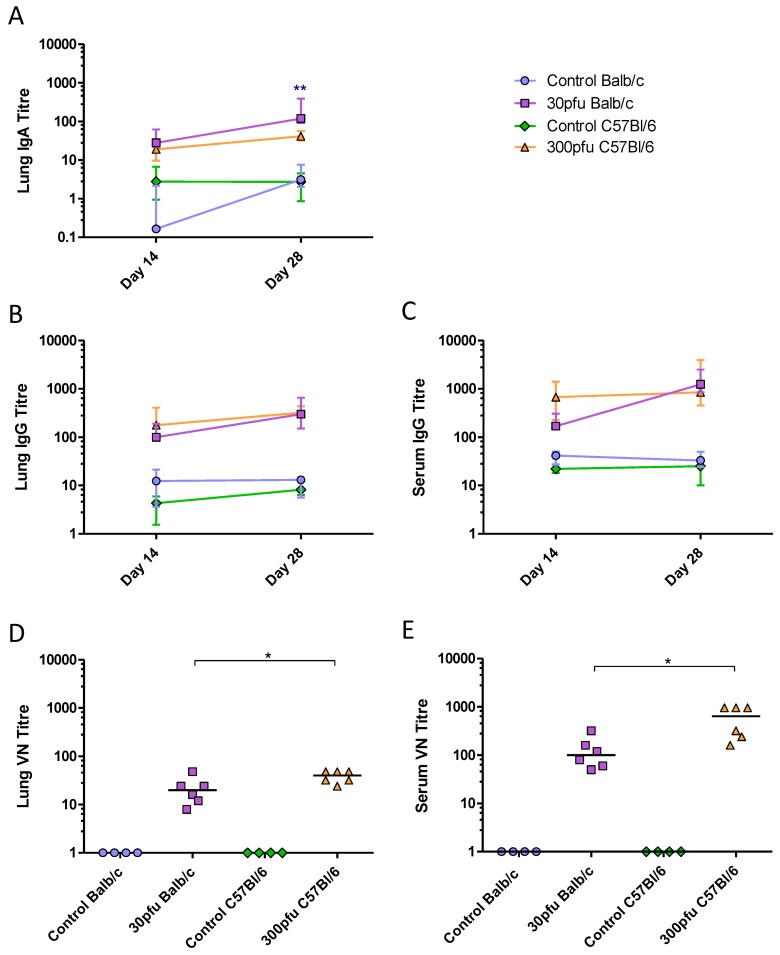
PVM-specific antibody response in the serum and lungs. Five to six week-old Balb/c and C57Bl/6 mice were inoculated with medium, 30 pfu (Balb/c) or 300 pfu (C57Bl/6) of PVM 15 and sacrificed on days 14, 28, and 42 p.i.. Time course of (**A**) lung IgA, (**B**) lung IgG, and (**C**) serum IgG, represented as the group median with vertical lines indicating the interquartile range. VN titres on day 28 in lung (**D**) and serum (**E**). Each data point represents a single animal and the line represents the group median. *, *p* <0.05; **, *p* <0.01.

To determine the magnitude and quality of the adaptive immune responses Balb/c and C57Bl/6 mice were inoculated intranasally with 50 µL of medium or a sub-lethal dose of PVM (30 pfu in Balb/c and 300 pfu in C57Bl/6), and followed for a longer period p.i.. To examine the PVM-specific humoral response, production of IgA, IgE, and IgG in the lungs and sera of Balb/c and C57Bl/6 mice was measured by ELISA. Neither strain produced IgE in the sera or lungs. IgA was detected in the lungs and IgG was present in the lungs and sera of both strains by day 14 p.i.. Balb/c mice showed increasing levels of IgG and IgA in the lungs, as well increasing IgG in the serum between days 14 and 28 p.i. (Figure 7A–C). Although they generally had the same or slightly lower levels of antibodies than Balb/c mice, the C57Bl/6 mice had already developed maximal antibody titres by day 14 p.i., which were maintained until day 28. To determine the biological relevance of the antibodies produced in these two strains of mice, a virus neutralization (VN) assay was performed (Figure 7D and E) showing that all PVM-infected mice contained VN antibodies in the serum and lungs on day 28 p.i.. Despite having similar levels of PVM-specific IgG in the serum and lower IgA levels in the lung, the C57Bl/6 mice had significantly higher VN antibody titres compared to the Balb/c mice. 

**Figure 8:**
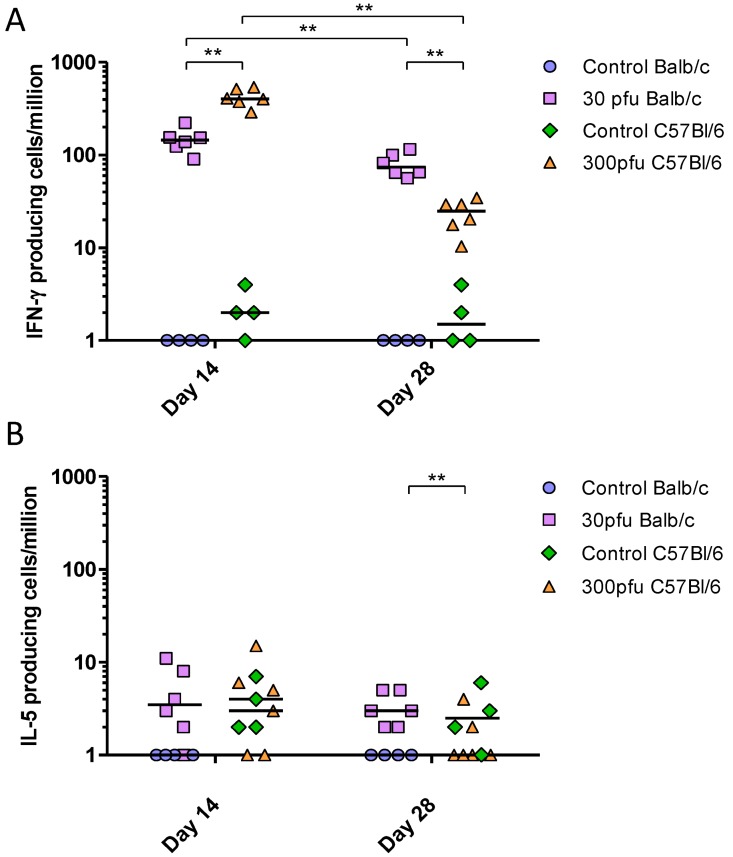
PVM-specific IFN-γ-secreting (**A**) and IL-5-secreting (**B**) splenocytes following *in vitro* restimulation with PVM-infected cell lysates. Five to six week-old Balb/c and C57Bl/6 mice were inoculated with medium, 30 pfu (Balb/c) or 300 pfu (C57Bl/6) and sacrificed on days 14, 28, and 42 p.i.. The PVM-specific response is calculated by subtracting the number of cytokine-secreting cells restimulated with mock-infected cell lysates from those restimulated with PVM-infected lysates. The data points represent individual animals, with the median indicated by a line. **, *p* <0.01.

To determine the T-helper bias of the immune response elicited by PVM in Balb/c and C57Bl/6 mice, we performed IFN-γ and IL-5 ELISPOT assays. All infected mice generated significantly higher numbers of PVM-specific IFN-γ-secreting cells compared to mock-infected animals of the same strain (Figure 8), while IL-5-secreting cells numbers remained low at all time points, suggesting a Th1-biased response to PVM in both strains of mice. However, the two strains showed a difference in the kinetics of the T-cell response. The C57Bl/6 mice showed a strong PVM-induced IFN-γ response as early as day 14, significantly higher than that of Balb/c mice; however, these responses were reduced between days 14 to 28 p.i., at which time they became lower than those in Balb/c mice. 

These data show that the adaptive immune response in both strains was Th1-biased. However, while the IgG and IgA titres in the lungs were overall similar between Balb/c and C57Bl/6 mice, the C57Bl/6 mice appeared to develop a more rapid systemic response based on higher serum IgG levels and IFN-γ secreting splenocytes on day 14 after infection. In addition, the C57Bl/6 mice had higher VN antibody levels in the lung and serum four weeks after infection. 

## 3. Experimental Section

### 3.1. Cell Lines and Virus

PVM 15 (American Type Culture Collection (ATCC)) was propagated in Baby Hamster Kidney (BHK)-21 cells (ATCC) in growth medium consisting of Dulbecco’s Modified Eagle Medium (DMEM; Sigma) supplemented with 2% fetal bovine serum (FBS, PAA Laboratories Inc), 0.1 mM non-essential amino acids (Invitrogen), 10 mM HEPES (Invitrogen), and 50 μg/mL gentamicin (Invitrogen). 

### 3.2. Challenge of Mice with PVM

Five to six week-old female Balb/c and C57Bl/6 mice (Charles River Laboratories) were housed in groups of four to six animals and acclimatized for one week. Mice were placed under light anaesthesia and inoculated intranasally with 50 µL of medium or with 30, 300, or 3000 pfu of PVM 15 in 50 µL. Mice were weighed daily and scored for clinical illness according to a modified version of Morton and Griffiths [42]. Sera were collected at regular intervals for detection of IgG. Mice were euthanized by an overdose of isoflurane and bled out by cardiac puncture. Spleens were collected for cytokine enzyme-linked immunospot (ELISPOT) assays. Lungs were collected for detection of cytokines/chemokines, virus, infiltrating cell populations, production of IgG and IgA, or histology. All experiments were performed within the guidelines of the Canadian Council for Animal Care.

### 3.3. Lung Samples

To evaluate virus replication and innate responses, lungs were collected daily from PVM-infected or mock-infected mice. The single-lobed, left lung was clamped off and removed into a 2 mL screwcap tube (VWR International) containing a ~1 mL volume of 2.4 mm zirconia beads (Biospec Products, Inc.) and 1.5 mL of DMEM supplemented as above, but with 1 × antibiotic/antimycotic (Invitrogen) and without FBS. The multi-lobed, right lung was removed into a tube containing 1 mL of Trizol^®^ reagent (Invitrogen) and 2.4 mm zirconia beads. The lungs were homogenized for 10 s at 4800 rpm using a Mini-beadbeater (Biospec Products, Inc.) and centrifuged at 4 °C for 1 min at 10,000 × g to remove the gross debris. All samples were flash-frozen in liquid nitrogen immediately following centrifugation, and stored at −80 °C. Prior to removal, the right lung was washed with 500 μl of phosphate buffered saline (PBS), pH 7.2 (Invitrogen) supplemented with 2% FBS and 50 μM EDTA, and the fluids from groups of four to six mice were pooled. Cytospin slides were prepared with 5 × 10^4^ and 1 × 10^5^ brochoalveolar lavage (BAL) cells per slide using a Cytospin 4 (Thermo Shandon). The slides were stained with a Giemsa-Wright stain (Bayer HealthCare) using an automated slide stainer, and differential analysis of the cell populations was performed by counting at least 200 cells in a blinded manner. 

### 3.4. Histology

For histology the multi-lobed right lung was collected on day 6 p.i. and perfused with 1 mL of 10% neutral buffered formalin (VWR) and placed in a cassette, which was then immersed in formalin. The perfused lungs were embedded in paraffin wax and sectioned. Duplicate 5 μm sections were stained with hematoxylin and eosin, and scored in a blinded manner by a veterinary pathologist. Scores were given based on the presence and severity, as well as the dissemination of lesions characterized by cellular infiltrates and edema in the tissue surrounding the bronchioles and blood vessels visible in lung sections. A score of 0 denotes a normal lung, 1 indicates signs of perivascular edema and mild perivasculitis, limited to small foci that were not widespread, while a score of 2 denotes moderate levels of perivasculitis, vasculitis, and multifocal perivascular edema. Finally, a score of 3 represents animals with extensive, severe, multifocal perivasculitis, vasculitis, and vascular edema, and/or necrotic and fibrinous areas. 

### 3.5. Lung Fragment Cultures

To measure local adaptive responses the multilobed lungs were collected and placed into a tube containing 5 mL of RPMI 1640 medium (Invitrogen) supplemented with 0.1 mM non-essential amino acids, 10 mM HEPES buffer, 1 mM sodium pyruvate (Invitrogen), 2 mM L-glutamine (Invitrogen), 1 × antibiotic/antimycotic (Invitrogen), 50 μg/mL gentamicin (Invitrogen), and 10% FBS (lung fragment culture (LFC) medium). The lungs were cut into four roughly equal-sized pieces, placed into four wells of a 48-well plate (Corning Inc.) containing LFC medium, and minced into pieces ~1 mm in diameter. The lung fragments were cultured for 5 days at 37 °C, at which point the culture medium was collected (BD Biosciences) and centrifuged at 4 °C and 311 × g, for 10 min. The supernatants were stored at −80 °C. 

### 3.6. Lung Cell Isolation and Flow Cytometry

The lungs were gently perfused via the right ventricle with 5 mL of Hanks balanced salt solution (HBSS) (Gibco) containing 0.5 mM EDTA. Lungs were then mechanically disrupted using a gentle MACS dissociator (Miltenyi Biotec) according to the manufacturer’s instructions and incubated with collagenase type IA (0.5 mg/mL; Sigma-Aldrich) and type IV bovine pancreatic DNase (20 µg/mL; Sigma-Aldrich) for 30 min at 37 °C in HBSS containing 5% FBS. After digestion, lung cells were filtered through a 100 µm nylon screen cell strainer. Single cell suspensions were washed, contaminating erythrocytes were lysed using ACK lysis buffer (Invitrogen), and viable cells were counted by trypan blue exclusion. To detect intracellular IFN-γ production in lung cells, cells were incubated for 6–8 h at 37 °C in complete RPMI containing 1 µL Golgi plug per mL. Subsequently, the cells were stained with the following antibodies from BD Biosciences: anti-CD8a FITC (cat no.553031), anti-CD3 PE (cat no.555275), anti-CD4 FITC (cat no.553729), anti-CD335(NKp46) PerCP-Cy™5.5 (cat no.560800) at 4 °C for 30 min, followed by permeabilization and fixation with Cytofix/Cytoperm™ Plus (BD Biosciences) at 4 °C for 20–30 min. Finally, cells were stained with anti–IFN-γ-APC (cat no.554413) from BD Biosciences at 4 °C for 30 min. Flow cytometry was performed using a FACS Calibur (BD Biosciences). Analysis of flow cytometry data was performed using Kaluza software.

### 3.7. PVM Quantification

PVM was quantified using a standard immunofluorescent plaque assay. Ten-fold serial dilutions of mouse lung homogenates were made in sterile 96-well dilution plates (Nalgen Nunc International) and 100 μl was transferred in duplicate onto 80% confluent BHK-21 cell monolayers. After 72 h incubation at 37 °C and 5% CO_2 _the cells were fixed with an ice-cold solution of 4 parts acetone to 1 part methanol, and dried at RT. The staining procedure was carried out at RT, and the plates were washed three times before blocking, and between each incubation in PBS. Plates were blocked for 30 min in PBS containing 5% goat serum (Invitrogen), followed by a 2–3 h incubation with a 1:500 dilution of a PVM nucleoprotein (N protein)-specific polyclonal rabbit antibody in PBS (made in-house) containing 1% goat serum, and a 1 h incubation in darkness with an Alexafluor488-congujated goat-anti-rabbit antibody (Invitrogen). The N-specific antibody was raised by immunization of rabbits with alternately KLH- and BSA-conjugated peptide (VVAKELKTGARLPDNQRHTAPDCGV) as described previously [43], and the specificity was confirmed by Western blotting (Supplementary Figure 1). Plaques were visualized ad counted using a fluorescent microscope (Zeiss). 

### 3.8. Analysis of Chemokine and Cytokine mRNA Expression

Following RNA isolation according to the manufacturer’s instructions (Invitrogen) and cDNA synthesis using a QuantiTect Reverse Transcription Kit (Qiagen), semi-quantitative real-time PCR (qPCR) was carried out using Platinum SYBR Green qPCR Supermix-UDG (Invitrogen) as per manufacturer’s instructions. The primers and annealing temperatures used in the qPCR experiments are listed in Table 1. Some primers were designed in house using NCBI PrimerBlast (http://www.ncbi.nlm.nih.gov/tools/primer-blast/) and primer design software (CloneManager Version 9.0). The qPCR reaction was carried out according to the following parameters: 40 cycles of denaturation at 95 °C for 30 s, followed by 30 s annealing and extension. Expression levels of cytokine and chemokine transcripts were calculated using the Bio-Rad analysis software (Bio-Rad CFX Manager Version 2.0), normalized against both β-actin and GAPDH, and expressed as the normalized fold-change over mock-infected control animals euthanized on the same day.

**Table 1:** Cytokine and Chemokine Primers and their Optimal Annealing Temperatures.

Target gene	Direction	Sequence	Source	Annealing Temp (°C)
β-actin	Forward	ACTGGGACGACATGGAG	[44]	57.5
	Reverse	GTAGATGGGCACAGTGTGGG		
GAPDH	Forward	AACTTTGGCATTGTGGAAGG	[45]	57.5
	Reverse	ACACATTGGGGGTAGGAACA		
CCL3/MIP-1α	Forward	CTTCTCTGTACCATGACACTC	[44]	57.5
	Reverse	AGGTCTCTTTGGAGTCAGCG		
CXCL8/MIP-2	Forward	TGCGCCCAGACAGAAGTCATAGC	designed in house	63.9
	Reverse	GCTCTAGAGTCAGTTAGCCTTGCCTTTG		
CCL2/MCP-1	Forward	CTTCTGGGCCTGCTGTTCA	[46]	57.5
	Reverse	CCAGCCTACTCATTGGGATCA		
IFN-γ	Forward	TCAAGTGGCATAGATGTGGAAGAA	[46]	57.5
	Reverse	TGGCTCTGCAGGATTTTCATG		
IL-4	Forward	GGAGATGGATGTGCCAAACG	designed in house	63.9
	Reverse	ACCTTGGAAGCCCTACAGAC		
IFN-α	Forward	CCTGTGTGATGCAACAGGTC	[47]	59.3
	Reverse	TCACTCCTCCTTGCTCAATC		
IFN-β	Forward	ATCATGAACAACAGGTGGATCCTCC	[47]	63.9
	Reverse	TTCAAGTGGAGAGCAGTTGAG		
TNF-α	Forward	GAACTGGCAGAAGAGGCACT	[47]	68.9
	Reverse	AGGGTCTGGGCCATAGAACT		
CXCL10/IP-10	Forward	GAGATCATTGCCACGATGAA	designed in house	63.9
	Reverse	CACTGGGTAAAGGGGAGTGA		
CCL5/RANTES	Forward	CTCACTGCAGCCGCCCTCTG	designed in house	57.5
	Reverse	CCTTGACGTGGGCACGAGGC		
CCL11/Eotaxin	Forward	AGAGGCTGAGATCCAAGCAG	designed in house	63.9
	Reverse	CAGATCTCTTTGCCCAACCT		

### 3.9. PVM-Specific ELISA

Immulon II plates (Thermo Electron) were coated with PVM-infected and mock-infected cell lysates. Plates were washed, and blocked with 5% gelatin in PBS (Sigma-Aldrich). Four-fold serial dilutions of serum or LFC supernatant were prepared in 96-well non-sterile dilution plates (Nalgen Nunc International), starting at a 1:40 dilution for serum and 1:10 for LFC supernatants. The diluted sera and LFC supernatants were added to the coated ELISA plates and incubated overnight at 4 °C. The plates were washed and either 1:5000 diluted AP-conjugated IgG (Kirkegaard & Perry Laboratories) or 1:2000 diluted biotinylated-anti-mouse IgA (Invitrogen) was added to the plates and allowed to react for 1–2 h. For the IgA ELISA, an additional 1 h incubation with a 1:10,000 dilution of AP-conjugated streptavidin (Jackson ImmunoResearch Laboratories) was carried out at RT. All washes were performed with PBS with 0.05% Tween 20 (Sigma-Aldrich) (PBST) and ddH_2_O. The plates were developed with p-nitrophenyl phosphate (Sigma-Aldrich) and the absorbances read at 405 nm with a reference wavelength of 490 nm. Titres were calculated based on the dilution at which the ELISA read-out for the PVM-positive antigen was equal to the highest reading from the same animal’s serum tested with the PVM-negative antigen. 

### 3.10. Virus Neutralization Assay

Sera and LFC supernatants were serially diluted in in sterile flat-bottom 96-well tissue culture plates (Nalgen Nunc International) starting at 1:10 and 1:2, respectively, and continuing in 2-fold dilutions down the plate. An equal volume of PVM 15 at a concentration of 500 pfu/well was added to the dilution plate, such that the resulting dilution of the serum or supernatant was 1:20 and 1:4, respectively. After 1 h incubation at 37 °C and 5% CO_2_, the virus mixture was transferred in duplicate onto BHK-21 cell monolayers grown to 80% confluency in flat-bottom 96-well tissue culture plates. Following a further 72 h incubation at 37 °C and 5% CO_2_, the cells were fixed and stained according to the PVM plaque assay protocol. 

### 3.11. IFN-γ and IL-5 ELISPOT Assays

Splenocytes were isolated as previously described [48]. Multiscreen-HA ELISPOT plates (Millipore) were coated with murine IFN-γ- or IL-5-specific monoclonal antibodies (BD PharMingen) at 2 μg/mL in sterile coating buffer. After overnight incubation at 4 °C, the plates were washed four times with sterile PBS, pH 7.2 (Invitrogen) and blocked for 1 h with 1% BSA (Sigma-Aldrich) at 37 °C. Splenocytes were restimulated with medium alone, mock- or PVM-infected cell lysates at 25 μg/mL or Concanavalin A (Con A). Each treatment was assayed in triplicate for each animal. After ~40 h of incubation at 37 °C in a 5% CO_2_ incubator, the medium was removed and the cells were lysed in ddH_2_O. Plates were washed three times in PBST and twice with ddH_2_O, and then biotinylated anti-mouse IFN-γ or IL-5 monoclonal antibodies (BD Biosciences) were added to the wells at a concentration of 2 μg/mL in PBS with 1% BSA. After 1–2 h incubation at RT, the plates were washed, and alkaline phosphatase (AP)-conjugated streptavidin was added at a dilution of 1:1000 in PBS with 1% BSA (Sigma-Aldrich) for 1-2 h at RT. After a final wash, cytokine-secreting cells were visualized by the addition of 5-bromo-4-chloro-3-indolylphosphate and nitroblue substrate (Sigma-Aldrich). Plates were washed in ddH_2_O and spots were counted under an inverted microscope (Olympus SZ71). The results were expressed as the number of cytokine-secreting cells per million splenocytes stimulated with mock-infected cell lysates subtracted from the number of cytokine-secreting cells stimulated with PVM-infected cell lysates. 

### 3.12. Statistical Analysis

Statistical software (GraphPad Prism Version 5.00) was used to analyze all data. Since there were few animals and the variability in biological systems makes normal distribution unlikely, the data were analyzed using non-parametric tests. The Kruskall-Wallis test was used to determine whether there was a difference between all groups. If the test indicated significant differences between the groups, Mann-Whitney U tests were used to compare the median of individual groups. Differences were considered significant if *p* < 0.05. 

## 4. Conclusions

The goal of this study was to compare the immune response of Balb/c and C57Bl/6 mice to PVM 15 in order to understand the relative susceptibility of these strains to infection. To our knowledge, this is the first direct comparison of the pathogenesis of PVM 15 in Balb/c and C57Bl/6 mice. Based on our results, it appears that a suppression or delay in the innate immune response, as well as decreased neutrophil and NK cell infiltration, in the C57Bl/6 strain could be a major factor in protecting these mice from severe illness. Furthermore, while both mouse strains developed a Th1-biased response to PVM infection, the VN antibody levels were higher in C57Bl/6 mice than in Balb/c mice, which correlated to an earlier increase in PVM-induced IFN-γ secreting cells. Surprisingly, neither strain developed eosinophilia following PVM 15 infection**.** During infection with PVM J3666, the inflammatory cell recruitment is of typically eosinophilic nature with early and prominent recruitment of eosinophils. Amongst the mouse strains documented by Anh and colleagues in their study all, except SJL, showed eosinophilic infiltration [12]. Furthermore, PVM J3666 infects murine eosinophils and elicits cytokine production further supporting the involvement of eosinophils in PVM J3666 pathogenesis [49]. In this respect strain PVM strain 15 differs from the J366 strain at least in Balb/c and C57Bl/6 mice, although it is not known whether PVM 15 infects eosinophils. 

These results suggest that in contrast to J3666, the PVM 15 strain has a distinct mode of pathogenesis in these two mouse strains. Balb/c mice responded to PVM 15 infection with earlier and higher expression of cytokines and chemokines, and earlier and enhanced lung inflammation dominated by neutrophils. These mice also showed earlier and higher levels of viral replication compared to C57Bl/6 mice given the same dose. Surprisingly, the IFN-α response was stronger in Balb/c mice than in C47Bl/6 mice. Although IFN-α usually suppresses viral replication, this was not the case in the PVM-infected Balb/c mice. This could be related to the fact that PVM, like RSV, suppresses the type-I IFN response [50], which might have been more efficient during production of high levels of de novo synthesized PVM proteins during viral replication. Alternatively, viral entry and replication of PVM in epithelial cells may be more efficient in Balb/c mice. The lungs can be partially protected by the immunomodulatory effects of surfactant proteins (SP) secreted by type II pneumocytes in the lower airways. The basal levels of surfactant A and D proteins are different in the lungs of naive Balb/c and C57Bl/6 mice, and are differentially upregulated upon sensitization with antigens [51], and as such may play a role in the different susceptibility of these two strains to PVM. Furthermore, naïve Balb/c mice have higher TLR4 mRNA expression in the lungs than C57Bl/6 mice, which may indicate a difference in TLR surface expression on resident macrophages or the respiratory epithelium [52] facilitating PVM entry. If Balb/c macrophages have higher levels of surface TLR4, this could trigger a stronger and earlier immune response to PVM infection than in C57Bl/6 mice. In a recent study by Glineur *et al.* on the potential mechanisms of the PVM resistance of SJL/J mice a role for innate, but not adaptive, immune responses was proposed, which would agree with our observations for PVM 15 in Balb/c and C57Bl/6 mice. They also concluded that PVM resistance of SJL/J mice is polygenic, that the resistance genes are recessive, and that radioresistant lung epithelial cells and macrophages may control the severity of lung disease caused by PVM [53]. A study of the contribution of epithelial cells and macrophages may provide further information on the pathogenesis of PVM 15 in Balb/c and C57Bl/6 strains. 

The Balb/c strain had a slower but ultimately stronger cell-mediated response to PVM, along with enhanced production of mucosal IgA by day 28; however, the antibody response was functionally less effective than that in the C57Bl/6 strain. Since RSV can cause a spectrum of disease depending on the age and immune history of the child in question, both mouse strains may be useful in examining specific aspects of pneumovirus pathogenesis. In terms of comparing their usefulness as a model of RSV, the next critical step will be to determine the protective efficacy of these responses upon reinfection. The C57Bl/6 strain infected with 300 pfu showed a rapid decline in activated peripheral T cells similar to that reported in infants monitored at the height of severe infection and four weeks later. Since humoral responses in children are also known to decline following infection with RSV, an analysis of the memory response to PVM in these two strains of mice will provide further insight into their use as a model of RSV.

## Supplementary Files

Supplementary File 1 Supplementary Figure (PDF, 81 KB)

## References

[1] Bonville C.A., Bennett N.J., Koehnlein M., Haines D.M., Ellis J.A., DelVecchio A.M, Rosenberg H.F., Domachowske J.B. (2006). Respiratory dysfunction and proinflammatory chemokines in the pneumonia virus of mice (PVM) model of viral bronchiolitis. Virology.

[2] Rosenberg H.F., Bonville C.A., Easton A.J., Domachowske J.B. (2005). The pneumonia virus of mice infection model for severe respiratory syncytial virus infection: Identifying novel targets for therapeutic intervention. Pharmacol. Ther..

[3] El-Hajje M.J., Lambe C., Moulin F., Suremain N., Pons-Catalano C., Chalumeau M., Raymond J., Lebon P., Gendrel D. (2008). The burden of respiratory viral disease in hospitalized children in Paris. Eu. Eur. J. Pediatr..

[4] Deshpande S.A., Northern V. (2003). The clinical and health economic burden of respiratory syncytial virus disease among children under 2 years of age in a defined geographical area. Arch. Dis. Child.

[5] Iwane M.K., Edwards K.M., Szilagyi P.G., Walker F.J., Griffin M.R., Weinberg G.A., Coulen C., Poehling K.A., Shone L.P., Balter S. (2004). Population-based surveillance for hospitalizations associated with respiratory syncytial virus, influenza virus, and parainfluenza viruses among young children. Pediatrics.

[6] Glezen W.P., Taber L.H., Frank A.L., Kasel J.A. (1986). Risk of primary infection and reinfection with respiratory syncytial virus. Am. J. Dis. Child.

[7] Hall C.B. (1982). Respiratory syncytial virus: its transmission in the hospital environment. Yale J. Biol. Med..

[8] Welliver R.C. (2003). Review of epidemiology and clinical risk factors for severe respiratory syncytial virus (RSV) infection. J. Pediatrics.

[9] Openshaw P.J., Tregoning J.S. (2005). Immune responses and disease enhancement during respiratory syncytial virus infection. Clin. Microbiol. Rev..

[10] Byrd L.G., Prince G.A. (1997). Animal models of respiratory syncytial virus infection. Clin. Infect. Dis..

[11] Easton A.J., Domachowske J.B., Rosenberg H.F. (2004). Animal pneumoviruses: Molecular genetics and pathogenesis. Clin. Microbiol. Rev.

[12] Anh D.B., Faisca P., Desmecht D.J. (2006). Differential resistance/susceptibility patterns to pneumovirus infection among inbred mouse strains. Am. J. Physiol. Lung Cell. Mol. Physiol..

[13] Rosenberg H.F., Domachowske J.B. (2008). Pneumonia virus of mice: severe respiratory infection in a natural host. Immunol. Lett..

[14] Krempl C.D., Lamirande E.W., Collins P.L. (2005). Complete sequence of the RNA genome of pneumonia virus of mice (PVM). Virus Gene..

[15] Domachowske J.B., Bonville C.A., Easton A.J., Rosenberg H.F. (2002). Differential expression of proinflammatory cytokine genes *in vivo* in response to pathogenic and nonpathogenic pneumovirus infections. J. Infect. Dis..

[16] Krempl C.D., Collins P.L. (2004). Reevaluation of the virulence of prototypic strain 15 of pneumonia virus of mice. J. Virol..

[17] Horsfall F.L., Hahn R.G. (1940). A latent virus in normal mice capable of producing pneumonia in its natural host. J. Exp. Med..

[18] Kuroda E., Kito T., Yamashita U. (2002). Reduced expression of STAT4 and IFN-gamma in macrophages from BALB/c mice. J. Immunol..

[19] Launois P., Swihart K.G., Milon G., Louis J.A. (1997). Early production of IL-4 in susceptible mice infected with Leishmania major rapidly induces IL-12 unresponsiveness. J. Immunol..

[20] Liu T., Matsuguchi T., Tsuboi N., Yajima T., Yoshikai Y. (2002). Differences in expression of toll-like receptors and their reactivities in dendritic cells in BALB/c and C57BL/6 mice. Infect. Immun..

[21] Scharton-Kersten T., Afonso L.C., Wysocka M., Trinchieri G., Scott P. (1995). IL-12 is required for natural killer cell activation and subsequent T helper 1 cell development in experimental leishmaniasis. J. Immunol..

[22] Srikiatkhachorn A., Chang W., Braciale T.J. (1999). Induction of Th-1 and Th-2 responses by respiratory syncytial virus attachment glycoprotein is epitope and major histocompatibility complex independent. J. Virol..

[23] Srikiatkhachorn A., Braciale T.J. (1997). Virus-specific CD8+ T lymphocytes downregulate T helper cell type 2 cytokine secretion and pulmonary eosinophilia during experimental murine respiratory syncytial virus infection. J. Exp. Med..

[24] Garvey T.L., Dyer K.D., Ellis J.A., Bonville C.A., Foster B., Prussin C., Easton A.J., Domachowske J.B., Rosenberg H.F. (2005). Inflammatory responses to pneumovirus infection in IFN-alpha beta R gene-deleted mice. J. Immunol..

[25] Bonville C.A., Lau V.K., DeLeon J.M., Gao J.L., Easton A.J., Rosenberg H.F., Domachowske J.B. (2004). Functional antagonism of chemokine receptor CCR1 reduces mortality in acute pneumovirus infection *in vivo*. J. Virol..

[26] Domachowske J.B., Bonville C.A., Gao J.L., Murphy P.M., Easton A.J., Rosenberg H.F. (2000). The chemokine macrophage-inflammatory protein-1 alpha and its receptor CCR1 control pulmonary inflammation and antiviral host defense in paramyxovirus infection. J. Immunol..

[27] Bonville C.A., Easton A.J., Rosenberg H.F., Domachowske J.B. (2003). Altered pathogenesis of severe pneumovirus infection in response to combined antiviral and specific immunomodulatory agents. J. Virol..

[28] Bonville C.A., Bennett N.J., Percopo C.M., Branigan P.J., Del Vecchio A.M., Rosenberg H.F., Domachowske J.B. (2007). Diminished inflammatory responses to natural pneumovirus infection among older mice. Virology.

[29] Domachowske J.B., Bonville C.A., Dyer K.D., Easton A.J., Rosenberg H.F. (2000). Pulmonary Eosinophilia and Production of MIP-1[alpha] Are Prominent Responses to Infection with Pneumonia Virus of Mice. Cellular Immunol..

[30] Rigaux P., Killoran K.E., Qiu Z., Rosenberg H.F. (2012). Depletion of alveolar macrophages prolongs survival in response to acute pneumovirus infection. Virology.

[31] Noah T.L., Becker S. (2000). Chemokines in nasal secretions of normal adults experimentally infected with respiratory syncytial virus. Clin. Immunol..

[32] Sheeran P., Jafri H., Carubelli C., Saavedra J., Johnson C., Krisher K., Sánchez P.J., Ramilo O. (1999). Elevated cytokine concentrations in the nasopharyngeal and tracheal secretions of children with respiratory syncytial virus disease. Pediatr. Infect. Dis. J..

[33] Abu-Harb M., Bell F., Finn A., Rao W.H., Nixon L., Shale D., Everard M.L. (1999). IL-8 and neutrophil elastase levels in the respiratory tract of infants with RSV bronchiolitis. Eur. Resp. J..

[34] Bont L., Heijnen C.J., Kavelaars A., van Aalderen W.M., Brus F., Draaisma J.T., Geelen S.M., van Vught H.J. (1999). Peripheral blood cytokine responses and disease severity in respiratory syncytial virus bronchiolitis. Eur. Respir. J..

[35] Bonville C.A., Percopo C.M., Dyer K.D., Gao J., Prussin C., Foster B., Rosenberg H.F., Domachowske J.B. (2009). Interferon-gamma coordinates CCL3-mediated neutrophil recruitment *in vivo*. BMC Immunol..

[36] Claassen E.A.W., van der Kant P.A.A., Rychnavska Z.S., van Bleek G.M., Easton A.J., van der Most R.G. (2005). Activation and Inactivation of Antiviral CD8 T Cell Responses during Murine Pneumovirus Infection. J. Immunol..

[37] Biron C.A., Nguyen K.B., Pien G.C., Cousens L.P., Salazar-Mather T.P. (1999). Natural killer cells in antiviral defense: function and regulation by innate cytokines. Annual Rev. Immunol..

[38] Campbell J.J., Qin S., Unutmaz D., Soler D., Murphy K.E., Hodge M.R., Wu L., Butcher EC. (2001). Unique subpopulations of CD56+ NK and NK-T peripheral blood lymphocytes identified by chemokine receptor expression repertoire. J. Immunol..

[39] Moretta A. (2002). Natural killer cells and dendritic cells: rendezvous in abused tissues. Nat. Rev. Immunol..

[40] Li F., Zhu H., Sun R., Wei H., Tian Z. (2012). Natural killer cells are involved in acute lung immune injury caused by respiratory syncytial virus infection. J. Virol..

[41] van Helden M.J., van Kooten P.J., Bekker C.P., Grone A., Topham D.J., Easton A.J., Boog C.J., Busch D.H., Zaiss D.M., Sijts A.J. (2012). Pre-existing virus-specific CD8(+) T-cells provide protection against pneumovirus-induced disease in mice. Vaccine.

[42] Morton D.B., Griffiths P.H. (1985). Guidelines on the recognition of pain, distress and discomfort in experimental animals and an hypothesis for assessment. Vet. Rec..

[43] Labiuk S.L., Babiuk L.A., van Drunen Littel-van den Hurk S. (2009). Major tegument protein VP8 of bovine herpesvirus 1 is phosphorylated by viral US3 and cellular CK2 protein kinases. J. Gen. Virol..

[44] Park J.K., Cho K., Johnson J., Perez R.V. (2004). Induction of MIP-1alpha in Kupffer cell by portal venous transfusion. Transplant. Immunol..

[45] Vogel C.F., Nishimura N., Sciullo E., Wong P., Li W., Matsumura F. (2007). Modulation of the chemokines KC and MCP-1 by 2,3,7,8-tetrachlorodibenzo-p-dioxin (TCDD) in mice. Arch. Biochem. Biophys..

[46] Overbergh L., Giulietti A., Valckx D., Decallonne R., Bouillon R., Mathieu C. (2003). The use of real-time reverse transcriptase PCR for the quantification of cytokine gene expression. J. Biomol. Tech..

[47] Pascual M., Fernandez-Lizarbe S., Guerri C. (2011). Role of TLR4 in ethanol effects on innate and adaptive immune responses in peritoneal macrophages. Immunol. Cell Biol..

[48] Mapletoft J.W., Latimer L., Babiuk L.A., van Drunen Littel-van den Hurk S. (2010). Intranasal immunization of mice with a bovine respiratory syncytial virus vaccine induces superior immunity and protection compared to those by subcutaneous delivery or combinations of intranasal and subcutaneous prime-boost strategies. Clin. Vaccine Immunol..

[49] Dyer K.D., Percopo C.M., Fischer E.R., Gabryszewski S.J., Rosenberg H.F. (2009). Pneumoviruses infect eosinophils and elicit MyD88-dependent release of chemoattractant cytokines and interleukin-6. Blood.

[50] Heinze B., Frey S., Mordstein M., Schmitt-Graff A., Ehl S., Buchholz U.J., Collins P.L., Staeheli P., Krempl C.D. (2011). Both nonstructural proteins NS1 and NS2 of pneumonia virus of mice are inhibitors of the interferon type I and type III responses *in vivo*. J. Virol..

[51] Atochina E.N., Beers M.F., Tomer Y., Scanlon S.T., Russo S.J., Panettieri R.A., Haczku A. (2003). Attenuated allergic airway hyperresponsiveness in C57BL/6 mice is associated with enhanced surfactant protein (SP)-D production following allergic sensitization. Respir. Res..

[52] Rodriguez-Martinez S., Cancino-Diaz M.E., Jimenez-Zamudio L., Garcia-Latorre E., Cancino-Diaz J.C. (2005). TLRs and NODs mRNA expression pattern in healthy mouse eye. British J. Ophthalmol..

[53] Glineur S., Tran Anh D.B., Sarlet M., Michaux C., Desmecht D. (2012). Characterization of the resistance of SJL/J mice to pneumonia virus of mice, a model for infantile bronchiolitis due to a respiratory syncytial virus. PloS One.

