# Supplementary material for: Innate and Adaptive Immune Response to Pneumonia Virus of Mice in a Resistant and a Susceptible Mouse Strain
Source: Viruses. 2013 Jan 21;5(1):295–320. doi: 10.3390/v5010295 (PMC3564122; doi:10.3390/v5010295)

Supplementary Figure 1. Western blotting of polyclonal antibodies specific for PVM N protein. Rabbits were injected with 0.5 mg keyhole limpet haemocyanin (KLH)-conjugated PVM N peptide (VVAKELKTGARLPDNQRHTAPDCGV) in Freund's complete adjuvant followed by two subsequent 0.3 mg booster immunizations, alternately with ovalbumin–peptide and KLH–peptide conjugates in Freund's incomplete adjuvant at 21-day intervals. The specificity of the antibody was confirmed based on reactivity with PVM-infected cell lysate or control lysate by Western blotting.

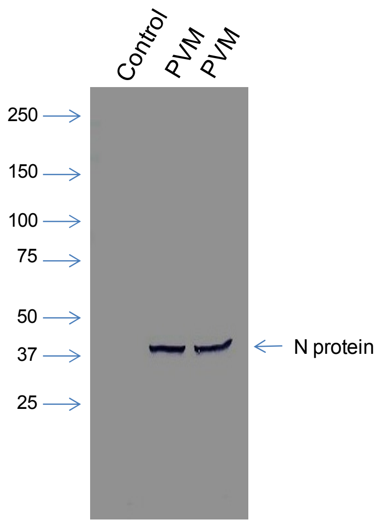

Supplement: Supplementary File 1 — Supplementary Figure (PDF, 81 KB) [file viruses-05-00295-s001.pdf]
